# In silico prioritization and further functional characterization of *SPINK1* intronic variants

**DOI:** 10.1186/s40246-017-0103-9

**Published:** 2017-05-04

**Authors:** Wen-Bin Zou, Hao Wu, Arnaud Boulling, David N. Cooper, Zhao-Shen Li, Zhuan Liao, Jian-Min Chen, Claude Férec

**Affiliations:** 1 Department of Gastroenterology, Changhai Hospital, Second Military Medical University, Shanghai, China; 2 Institut National de la Santé et de la Recherche Médicale (INSERM), U1078 Brest, France; 3 Etablissement Français du Sang (EFS)–Bretagne, Brest, France; 4 Shanghai Institute of Pancreatic Diseases, Shanghai, China; 5 Institute of Medical Genetics, School of Medicine, Cardiff University, Cardiff, UK; 6 Faculté de Médecine et des Sciences de la Santé, Université de Bretagne Occidentale (UBO), Brest, France; 7 Laboratoire de Génétique Moléculaire et d’Histocompatibilité, Centre Hospitalier Universitaire (CHU) Brest, Hôpital Morvan, Brest, France

**Keywords:** Aberrant mRNA transcripts, Chronic pancreatitis, In silico, Intronic variants, Non-canonical splice sites, Quantitative RT-PCR analysis, *SPINK1*, Splicing phenotype prediction

## Abstract

**Background:**

*SPINK1* (serine protease inhibitor, kazal-type, 1), which encodes human pancreatic secretory trypsin inhibitor, is one of the most extensively studied genes underlying chronic pancreatitis. Recently, based upon data from qualitative reverse transcription-PCR (RT-PCR) analyses of transfected HEK293T cells, we concluded that 24 studied *SPINK1* intronic variants were not of pathological significance, the sole exceptions being two canonical splice site variants (i.e., c.87 + 1G > A and c.194 + 2T > C). Herein, we employed the splicing prediction tools included within the Alamut software suite to prioritize the ‘non-pathological’ *SPINK1* intronic variants for further quantitative RT-PCR analysis.

**Results:**

Although our results demonstrated the utility of in silico prediction in classifying and prioritizing intronic variants, we made two observations worth noting. First, we established that most of the prediction tools employed ignored the general rule that GC is a weaker donor splice site than the canonical GT site. This finding is potentially important because for a given disease gene, a GC variant donor splice site may be associated with a milder clinical manifestation. Second, the non-pathological c.194 + 13T > G variant was consistently predicted by different programs to generate a new and viable donor splice site, the prediction scores being comparable to those for the physiological c.194 + 2T donor splice site and even higher than those for the physiological c.87 + 1G donor splice site. We do however provide convincing in vitro evidence that the predicted donor splice site was not entirely spurious.

**Conclusions:**

Our findings, taken together, serve to emphasize the importance of functional analysis in helping to establish or refute the pathogenicity of specific intronic variants.

**Electronic supplementary material:**

The online version of this article (doi:10.1186/s40246-017-0103-9) contains supplementary material, which is available to authorized users.

## Background

*SPINK1* (serine protease inhibitor, kazal-type, 1; OMIM #167790), which encodes pancreatic secretory trypsin inhibitor, is one of the most extensively studied genes underlying chronic pancreatitis [1]. Of the some 90 different nucleotide sequence variants listed in the *Chronic Pancreatitis Genetic Risk Factors Database* (http://www.pancreasgenetics.org/index.php; accessed 2 Jan 2017), 31 (34%) are intronic, a difficult category of sequence variant to ascertain in terms of their potential pathological relevance. Recently, using a ‘maxigene’ expression assay for which the full-length *SPINK1* genomic sequence (approximately 7 kb stretching from the translational initiation codon to the stop codon of the four-exon gene) was cloned into the pcDNA3.1/V5-His-TOPO vector, we analyzed the functional consequences of 24 *SPINK1* intronic variants for the mRNA splicing phenotype in transfected HEK293T cells by means of reverse transcription-PCR (RT-PCR) analysis. Based upon the observed splicing patterns, we concluded that none of the studied variants, apart from the two canonical splice site variants (i.e., c.87 + 1G > A and c.194 + 2T > C), were of pathological significance [2, 3].

However, upon reflection, we felt that whereas our conclusions regarding the two canonical splice site variants were solid, those relating to the other 22 intronic variants could have been too hasty. For example, some of these 22 intronic variants might have caused aberrant splicing albeit to a limited extent. However, such aberrantly spliced transcripts may have been rapidly degraded by the cellular mRNA quality control system as compared with the correctly spliced transcripts, resulting in a quantitative decrease in terms of the correctly spliced transcripts. To explore this possibility, we employed the commonly used in silico splicing prediction programs to prioritize these *SPINK1* intronic variants for further quantitative RT-PCR analysis.

## Materials and methods

### In silico splicing prediction

All 24 of the *SPINK1* intronic variants previously analyzed by the maxigene assay [2, 3] were re-examined in the context of in silico splicing prediction by means of Alamut® Visual version 2.7.1 (Interactive Biosoftware, Rouen, France) that included five prediction algorithms viz. SpliceSiteFinder-like, MaxEntScan, NNSPLICE, GeneSplicer, and Human Splicing Finder. We focused exclusively on the potential impact of the *SPINK1* intronic variants in terms of the disruption of known splice sites or the creation of new potential splice sites. We firstly used data derived from the two canonical splice site variants (i.e., c.87 + 1G > A and c.194 + 2T > C) as a first means to assess the performance of each of the five prediction programs. We then used the selected programs to prioritize variants for quantitative RT-PCR analysis.

### Quantitative RT-PCR analysis of four prioritized variants in the context of a maxigene assay

Four variants (i.e., c.87 + 363A > G, c.194 + 13T > G, c.194 + 1504A > G, and c.195-323C > T) were prioritized for quantitative RT-PCR analysis. The wild-type and variant expression vectors harboring the corresponding full-length genomic *SPINK1* genes have been previously described [2, 3]. HEK293T cell culture, transfection, reverse transcription, and real-time quantitative RT-PCR analyses were performed as described [4], except that the primer pair used for amplifying the full-length target gene transcripts was changed to 5′-GGAGACCCAAGCTGGCTAGT-3′ (forward) and 5′-AGACCGAGGAGAGGGTTAGG-3′ (reverse); the forward and reverse primers are located within the pcDNA3.1 5′- and 3′-untranslated regions, respectively (i.e., the primer pair Q1 as described in [5]).

### Further analyses of the c.194 + 13T > G variant in the context of a maxigene assay

#### Analysis of nonsense-mediated mRNA decay (NMD)

This analysis was performed as described in [4], with the *CEL-HYB1* and *CEL-HYB2a* expression vectors being replaced by the aforementioned full-length *SPINK1* wild-type and c.194 + 13T > G variant gene expression vectors, respectively.

#### Identification of the in silico predicted aberrant transcript

The c.194 + 13T > G variant was consistently predicted by the selected four programs to create a putative splice donor site (see Results and discussion). An allele-specific forward primer, 5′-ATGTTTTGAAAATCGGTGAGTAC-3′, was used together with the reverse primer of the aforementioned primer pair Q1 [5] to amplify this predicted aberrant transcript. The PCR program comprised an initial denaturation at 95 °C for 15 min, followed by 35 cycles of denaturation at 94 °C for 45 s, annealing at 58 °C for 45 s, and extension at 72 °C for 1 min.

#### Estimation of the frequency of the aberrant transcripts relative to the wild-type transcripts

Using the aforementioned primer pair Q1, we performed RT-PCR to amplify the full-length transcripts prepared from the c.194 + 13T > G variant maxigene-transfected HEK293T cells treated with cycloheximide. After addition of 3′-A overhangs, the purified products were cloned into the pcDNA3.1/V5-His-TOPO vector (Invitrogen, Carlsbad, CA, USA). Transformation was performed using XL10-Gold Ultracompetent Cells (Stratagene, La Jolla, CA, USA), and transformed cells were spread onto LB agar plates with 50 mg/mL ampicillin and were incubated at 37 °C overnight [4]. Bacterial colonies were picked to be added into a 25 μl PCR mixture, which contained 12.5 μl HotStarTaq Master Mix (QIAGEN), 0.4 μM each primer (i.e., primer pair C1 in [5]; the forward and reverse primer sequences are located within the beginning and the end regions of the *SPINK1* coding sequence, respectively). The PCR had an initial denaturation step at 95 °C for 15 min, followed by 30 cycles of denaturation at 94 °C for 45 s, annealing at 58 °C for 45 s, and extension at 72 °C for 45 s. The samples showing the expected band on the gel were cleaned and sequenced using the forward primer.

### Further analyses of the c.194 + 13T > G variant in the context of a minigene assay

#### Minigene construction and mutagenesis

A 567-bp fragment spanning the exon 3 of *SPINK1* as well as 230 bp flanking intronic sequences on both sides (Additional file 1) was amplified using primers 5′-CGGGCCCCCCCTCGAGTTTCAGAAGGGCCATAGGAC-3′ (forward) and 5′-TAGAACTAGTGGATCCCCAAGCTATCGACTATTTTGCTG-3′ (reverse). PCR was performed in a 25 μl reaction mixture containing 0.5 U KAPA HiFi HotStart DNA Polymerase, 5 μl 5× KAPA HiFi Buffer, 0.75 μl dNTP Mix, 20 ng expression vector containing the full-length wild-type *SPINK1* genomic sequence [5], and 0.3 μM each primer. The PCR program comprised an initial denaturation at 95 °C for 5 min followed by 30 cycles of denaturation at 98 °C for 20 s, annealing at 66 °C for 15 s, extension at 72 °C for 15 s, and a final extension at 72 °C for 5 min. The PCR products were cloned into the Exontrap vector pET01 (MoBiTec) that was linearized by restriction enzymes *BamH*I and *Xho*I, using the In-Fusion® HD Cloning kit (Clontech) in accordance with the manufacturer’s instructions. The resulting expression vector was termed the wild-type *SPINK1* exon 3 minigene.

The c.194 + 13T > G variant was introduced into the wild-type *SPINK1* exon 3 minigene as previously described for introducing the same variant into the wild-type *SPINK1* maxigene vector [2], except that the extension time was reduced from 13 to 5 min. The successful introduction of the variant was confirmed by DNA sequencing using primers 5′-GTAGCTGCCAGGAAGGAGTG-3′ (forward) and 5′-GGCCTCCAAAACCTACACAT-3′ (reverse).

#### Qualitative and quantitative RT-PCR analyses

HEK293T cell culture, transfection, and reverse transcription were performed as previously described [2]. Qualitative RT-PCR was performed in a 25 μl mixture containing 12.5 μl HotStarTaq Master Mix (QIAGEN), 0.4 μM each primer (5′-GAGGGATCCGCTTCCTGGCCC-3′ (forward) and 5′-CTCCCGGGCCACCTCCAGTGCC-3′ (reverse)), and 1 μl cDNA. The program had an initial denaturation at 95 °C for 15 min followed by 30 cycles of denaturation at 94 °C for 45 s, annealing at 58 °C for 45 s, and extension at 72 °C for 2 min. PCR products were cleaned by ExoSAP-IT (Affymetrix) and were sequenced using a BigDye Terminator v1.1 Cycle Sequencing Kit (Applied Biosystems).

Quantitative RT-PCR analysis was done as previously described [4], except that the primers used for target gene amplification were changed to those used for the aforementioned qualitative RT-PCR analysis.

## Results and discussion

The increasingly routine use of whole genome sequencing has led to the discovery of an increasing number of rare variants, particularly intronic variants, owing to the relatively large size of intronic sequences as compared to the exonic sequences of protein-coding genes. It is, in practice, generally unrealistic to functionally analyze the large number of intronic variants detected in protein-coding genes. In silico prediction is therefore commonly used both to classify and prioritize intronic variants for further functional analysis [6, 7]. Herein, we adopted this approach to prioritize *SPINK1* intronic variants, which had been previously classified as ‘non-pathological’ in accordance with data obtained from qualitative RT-PCR analyses of transfected HEK293T cells [2, 3], for further quantitative analysis. To this end, we predicted the impact on mRNA splicing of all 24 previously functionally analyzed *SPINK1* intronic variants by means of the widely used Alamut software suite, focusing on their potential disruption or creation of splice sites in accordance with previous studies [7, 8]. The corresponding score changes for each variant, predicted by each of the five prediction programs included within Alamut, are summarized in Additional file **2**.

### Using data from two canonical splice site variants as a first means to assess the relative performance of the five splicing prediction programs

There are currently no consensus guidelines as to which prediction tools should be used or how the results of predictions should be interpreted [9]. Importantly, of the 24 *SPINK1* intronic variants studied here, c.87 + 1G > A and c.194 + 2T > C affected canonical splice donor splice sites and were the only tested variants that had been previously shown to result in aberrant splicing using a maxigene assay [2, 3]. We therefore used data from these two variants as a means to assess the relative performance of the five splicing prediction programs included within the Alamut software suite.

#### GeneSplicer was excluded from consideration owing to its poor performance in relation to the two corresponding wild-type alleles

We first assessed the relative performance of the five splicing prediction programs by evaluating their prediction scores with respect to the corresponding wild-type alleles of the two canonical splice site variants. SpliceSiteFinder-like, MaxEntScan, NNSPLICE, and Human Splicing Finder all yielded scores that were ≥79.8% of their respective maxima. However, GeneSplicer only yielded a score of <27% of its maximum (Additional file 3) and was therefore excluded from further consideration.

#### A particular observation with respect to the c.194 + 2T > C variant

We then correlated the prediction scores from the four selected programs and the known splicing phenotypes in relation to the two canonical splice site variant alleles. In the context of the c.87 + 1G > A variant allele, SpliceSiteFinder-like, MaxEntScan, NNSPLICE, and Human Splicing Finder all predicted a score of zero (Table 1). This prediction was consistent with the complete exon 2 skipping that was observed in the maxigene assay [2].

**Table 1:** In vitro observed and in silico predicted mRNA splicing phenotypes associated with the two canonical splice site variants and four intronic variants prioritized for quantitative RT-PCR analysis

Intron	*SPINK1* variant	SpliceSiteFinder-like (0–100)	MaxEntScan (0–12)	NNSPLICE (0–1)	Human Splicing Finder (0–100)	In vitro observed mRNA splicing phenotype^a^
Canonical splice donor site variants						
2	c.87 + 1G > A	dss 79.8 → 0	dss 8.3 → 0	dss 0.9 → 0	dss 84.1 → 0	Complete exon 2 skipping
3	c.194 + 2T > C	dss 82.6 → 72.3	dss 11.1 → 0	dss 1.0 → 0	dss 92.1 → 0	Partial exon 3 skipping
Variants prioritized for quantitative RT-PCR analysis						
2	c.87 + 363A > G	−	−	−	dss 0 → 65.5	Normal
					ass 0 → 83.3	
3	c.194 + 13T > G	dss 0 → 82.0	dss 0 → 9.5	dss 0 → 0.9	dss 0 → 86.9	Normal
3	c.194 + 1504A > G	dss 0 → 77.2	−	dss 0 → 0.7	dss 0 → 83.2	Normal
3	c.195-323C > T	−	dss 0 → 6.3	dss 0 → 0.7	dss 0 → 75.1	Normal

*Abbreviations*: *dss* donor splice site, *ass* acceptor splice site

^a^In accordance with Zou et al. [2, 3]

A particular observation was made in the context of the c.194 + 2T > C variant. This variant was shown to lead to two distinct transcripts, one wild-type, the other lacking exon 3, the aberrant transcript being expressed at a much higher level than the wild-type transcript in our maxigene assay [2]. These results not only concur with those obtained from the analysis of mRNA derived from stomach tissue (in which *SPINK1* is also abundantly expressed) from a c.194 + 2T > C homozygote [10] but are also consistent with the general rule that GC is a lesser frequently used and weaker splice donor site as compared to the canonical GT site [11, 12]. However, only SpliceSiteFinder-like predicted a consistent reduced score (from 82.6 to 72.3) whilst MaxEntScan, NNSPLICE, and Human Splicing Finder all predicted a score of zero (Table **1**).

Taken together, in the context of the two canonical splice site variants, a good correlation was noted between the predictions of SpliceSiteFinder-like, MaxEntScan, NNSPLICE, and Human Splicing Finder and the in vitro maxigene-obtained functional results, although only SpliceSiteFinder-like yielded a perfect correlation in both cases.

### In silico prioritization of the 22 ‘non-pathological’ variants for further quantitative analysis

We classified the 22 empirically tested ‘non-pathological’ *SPINK1* intronic variants into three categories in accordance with the predictions by SpliceSiteFinder-like, MaxEntScan, NNSPLICE, and Human Splicing Finder (Additional file 2).

Category 1 comprised 13 variants, none of which were predicted to affect splicing by any of the four programs. These variants were excluded from further functional analysis.

Category 2 comprised three variants that were only predicted (and only by Human Splicing Finder) to disrupt a putative splice acceptor or donor site (i.e., c.88-352A > G, c.194 + 90A > T, and c.194 + 184 T > A). These predictions were clearly inappropriate because (i) the *SPINK1* gene is not known to have alternative transcripts and (ii) we did not observe any alternative transcripts from the wild-type *SPINK1* gene in the maxigene assay. They were therefore also excluded from further functional analysis.

Category 3 comprised 6 variants, each being predicted by at least one program to create a new donor or acceptor splice site. Of these, c.194 + 13T > G was predicted by all four programs to create a potential donor splice site and both c.194 + 1504A > G and c.195-323C > T were predicted by three of the four programs to create a potential donor splice site. These three variants were selected for further functional analysis (Table 1).

The remaining three category 3 variants were all predicted by one and the same program (i.e., Human Splicing Finder) to create a new donor or acceptor splice site (i.e., c.87 + 363A > G, c.195-1570C > A, and c.195-1399G > A). Of these, we selected that which had the highest predicted score (i.e., 83.3 of the maximum score of 100; c.87 + 363A > G), for further analysis (Table 1).

### All four prioritized variants were not found to significantly reduce mRNA expression by quantitative RT-PCR analysis

**Fig. 1:**
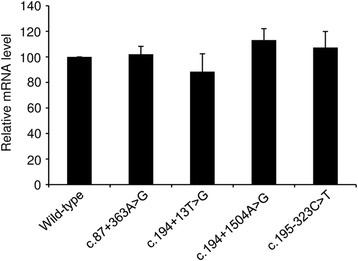
Relative mRNA expression levels associated with four variant *SPINK1* gene constructs compared to that of the wild-type, as determined by the quantitative RT-PCR analysis of HEK293T cells transfected with various maxigene expression constructs. Results are given as the mean ± SD from three independent transfection experiments. No statistically significant difference in mRNA expression level was noted between any of the variants and the wild-type

We performed quantitative RT-PCR analysis of the HEK293T cells co-transfected with each of the expression vectors harboring the respective full-length variant *SPINK1* genes and the reference PGL3-GP2 plasmid [4, 5]. We did not however observe statistically significant differences in mRNA expression level between any of the four variants and the wild-type (Fig. 1).

### Further analyses of the c.194 + 13T > G variant

Of the four variants subjected to quantitative RT-PCR analysis, c.194 + 13T > G was the only one that was consistently predicted by the SpliceSiteFinder-like, MaxEntScan, NNSPLICE, and Human Splicing Finder programs to create a potential donor splice site, with each program predicting a relatively high score (Table 1). Indeed, the predicted scores for this variant were even higher than, or at least equal to, the corresponding ones for the physiological c.87 + 1G donor splice site (Table 1). Moreover, the predicted scores for this variant were comparable to those for the physiological c.194 + 2T donor splice site (Table 1), the two sites being separated by only 10 bp.

**Fig. 2:**
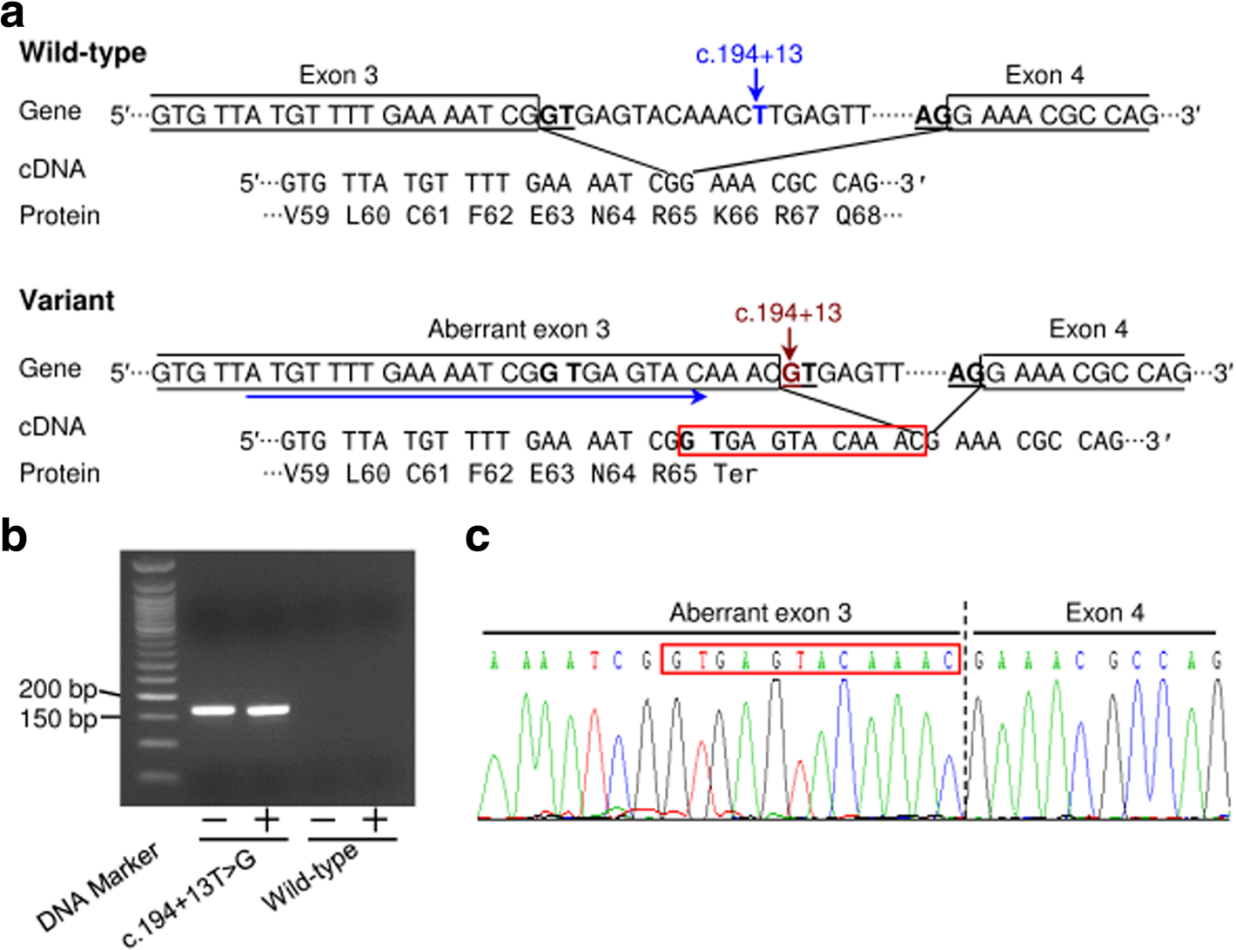
Confirmation of the Alamut-predicted creation of a new splice donor site by the *SPINK1* c.194 + 13T > G variant. **a** Schemas for the splicing of intron 3 with respect to the wild-type and variant alleles of c.194 + 13T > G. The splice donor (GT) and splice acceptor (AG) signals potentiating the normal and aberrant splicing of intron 3 are highlighted in *bold* and *underlined*. The *rightward pointing blue arrow* indicates the forward allele-specific primer designed to amplify the predicted aberrant transcript (as shown in **b**). The 12 bp intronic sequence inappropriately included within the predicted aberrant transcript is indicated by a *red box*. The amino acid sequences of the wild-type and predicted mutant proteins are also shown. **b** PCR identification of the aberrant transcripts expressed from the HEK293T cells transfected with the c.194 + 13T > G variant-containing maxigene expression construct. The primers used for amplification were the forward allele-specific primer as illustrated in (**a**) and a reverse primer located within the 3′ untranslated region of the expression vector. No PCR products were identified in cells transfected with the wild-type maxigene expression vector. *Plus* and *minus* symbols refer to cells treated with and without cycloheximide, respectively. **c** Sequence of the c.194 + 13T > G/+ PCR products as illustrated in (**b**). The 12 bp intronic sequence included within the aberrant transcript is indicated by a *red box*

The use of the predicted splice donor site would lead to the generation of an aberrant transcript containing a premature stop codon (Fig. 2a). Such a transcript might be subject to significant degradation by NMD [13], leading to a reduced mRNA expression level. In this regard, the aforementioned quantitative RT-PCR analyses hinted at a possible reduced level of expression of the correctly spliced transcripts from the c.194 + 13T > G variant maxigene (Fig. 1). To clarify this issue, we performed further quantitative RT-PCR analysis of the c.194 + 13T > G variant maxigene-transfected HEK293T cells with or without treatment with cycloheximide (a known NMD inhibitor [14]) as previously described [4], but no statistically significant changes were observed (Additional file 4). Additionally, we analyzed the impact on splicing of the c.194 + 13T > G variant in the context of a minigene assay. We found only a single band whose size was similar to that of the wild-type (Additional file 5a) and whose identity to the wild-type sequence was confirmed by sequencing the RT-PCR product. We further performed quantitative RT-PCR analysis of the HEK293T cells co-transfected with the c.194 + 13T > G variant-containing minigene expression vector and the PGL3-GP2 plasmid. We did not observe statistically significant differences in mRNA expression level between the c.194 + 13T > G variant and the wild-type (Additional file 5b).

We then speculated that such an aberrant transcript might exist but at such an extremely low level as compared to the correctly spliced transcript that it would be beyond the detection limit of quantitative RT-PCR analysis. We therefore designed an allele-specific forward primer (rightward blue arrow in Fig. 2a) in an attempt to detect this aberrant transcript if it were to exist. Use of this forward primer and a reverse primer located within the pcDNA3.1 3′-untranslated region succeeded in detecting a specific RT-PCR product from the c.194 + 13T > G variant maxigene-transfected HEK293T cells (both with and without cycloheximide treatment) but not in the wild-type *SPINK1* maxigene-transfected HEK293T cells (Fig. 2b). Sequencing of this specific RT-PCR product confirmed the use of predicted novel splice donor site (Fig. 2c).

To obtain an approximate estimate of the expression level of the aberrant transcript relative to the correctly spliced transcript, we then performed colony PCRs followed by sequencing of the full-length transcripts amplified from the c.194 + 13T > G variant maxigene-transfected HEK293T cells treated with cycloheximide. Sequencing of 100 PCR products of expected size revealed only wild-type transcript. [note that the wild-type and aberrant transcripts are indistinguishable by gel analysis owing to their length difference of only 12 bp; see Fig. 2a.] This suggested that the c.194 + 13T > G variant resulted in less than 1% of aberrantly spliced transcripts relative to the amount of wild-type transcript.

## Conclusions

In silico prioritization and subsequent quantitative RT-PCR analyses of selected *SPINK1* intronic variants for further functional characterization in a maxigene assay supported our previous classification of 24 *SPINK1* intronic variants as having pathological relevance (or not) in chronic pancreatitis [2, 3]. As in many studies, our results demonstrated the utility of in silico prediction in classifying and prioritizing intronic variants. However, we made two observations worth noting during this study. First, we found that most of the prediction programs included within the commonly used Alamut software suite ignore the general rule that GC is a weaker donor splice site as compared with the canonical GT donor splice site. This finding serves to remind us of a key point in medical genetics: in a given disease gene, a C introduced into the second position of a canonical GT donor splice site may have a milder clinical manifestation than a G or A. Second, the non-pathological c.194 + 13T > G variant was consistently predicted by the selected four programs to generate a potential donor splice site; the prediction scores being even higher than the physiological c.87 + 1G splice site. However, by means of allele-specific PCR, we provided convincing in vitro evidence that the predicted donor splice site was not entirely spurious (Fig. 2). These findings, taken together, serve to emphasize the importance of functional analysis in helping to establish or refute the pathogenicity of certain intronic variants, an issue of increasing importance in the new age of precision medicine [15, 16]. This notwithstanding, it should be pointed out that in the context of the c.194 + 13T > G variant, it would be desirable to investigate its effect in the corresponding carrier’s pancreatic tissue. However, obtaining such a tissue sample would be extremely difficult particularly given that the c.194 + 13T > G variant has so far been reported only once [17].

## Additional files

Additional file 1: Figure S1. The *SPINK1* sequence cloned into the Exontrap vector pET01. (PDF 251 kb)

Additional file 2: Table S1. In vitro observed and in silico predicted mRNA splicing phenotypes associated with the 24 *SPINK1* intronic variants under study. (PDF 110 kb)

Additional file 3: Figure S2. Alamut-predicted impact of the *SPINK1* c.87 + 1G > 1, c.194 + 2T > C, and c.194 + 13T > G variants on the disruption or creation of splice sites. (PDF 418 kb)

Additional file 4: Figure S3. Relative mRNA expression levels of the *SPINK1* c.194 + 13T > G variant-containing maxigene in transfected HEK293T cells in the presence (gray) and absence (black) of cycloheximide as determined by quantitative RT-PCR analysis. (PDF 225 kb)

Additional file 5: Figure S4. Further analyses of the *SPINK1* c.194 + 13T > G variant in a minigene assay. (PDF 184 kb)

## Abbreviations

ass: Acceptor splice site
dss: Donor splice site
NMD: Nonsense-mediated mRNA decay
RT-PCR: Reverse transcription-PCR
