# Supplementary material for: In silico prioritization and further functional characterization of SPINK1 intronic variants
Source: Hum Genomics. 2017 May 4;11:7. doi: 10.1186/s40246-017-0103-9 (PMC5418720; doi:10.1186/s40246-017-0103-9)
Supplement: Supplementary file 1 — The SPINK1 sequence cloned into the Exontrap vector pET01. (PDF 251 kb) [file 40246_2017_103_MOESM1_ESM.pdf]

```
TTTCAGAAGGGCCATAGGACTTACTAATGTCACACAGCTTAGAAATAGCAGAGGCATGACTTAAAACAAGGTTTT
CTGTCTCCAGATAGTAGGTTATTTCTCTTACAACACACAGTATCATTCTCCCAATCACAGTTATTCCCCAGAGAA
ATAAAACCATTTCAGAGATTTTGCTATGAACTCAAGAATGGAGAATAATGGGAAATGATTCTGTTTAATTCCATT
TTTAGGCCAAATGTTACAATGAACTTAATGGATGCACCAAGATATATGACCCTGTCTGTGGGACTGATGGAAATA
CTTATCCCAATGAATGCGTGTTATGTTTTGAAAATCGGTGAGTACAAACTTGAGTTTCTTTTAAACTATATATTT
TAAGTTAGTTATCTTCAAGTGTACTGATAATATGAATCTCACCCCGAGAAAAGCAAACTATTTACTTTTTCCAAA
AACAGTTATCTCTTTCTTATTCTCCCTTTTATATATTTAGCATTAAATATTATTTTTTAGAAGTCACTTGTATGA
TAAAAGCCTATATTTTTTACAGCAAAATAGTCGATAGCTTGG
```

Figure S1 The *SPINK1* sequence cloned into the Exontrap vector pET01. Shaded is exon 3 of the *SPINK1* gene.
