# Supplementary material for: In silico prioritization and further functional characterization of SPINK1 intronic variants
Source: Hum Genomics. 2017 May 4;11:7. doi: 10.1186/s40246-017-0103-9 (PMC5418720; doi:10.1186/s40246-017-0103-9)
Supplement: Supplementary file 2 — In vitro observed and in silico predicted mRNA splicing phenotypes associated with the 24 SPINK1 intronic variants under study. (PDF 110 kb) [file 40246_2017_103_MOESM2_ESM.pdf]

Table S1 *In vitro* observed and *in silico* predicted mRNA splicing phenotypes associated with the 24 *SPINK1* intronic variants under study

Intron	Variant	SpliceSiteFinder-like (0-100)	MaxEntScan (0-12)	NNSPLICE (0-1)	GeneSplicer (0-15)	Human Splicing Finder (0-100)	Functional analysis of mRNA splicing phenotype[a] (category[b])
1	c.56-619T>C	–	–	–	–	–	Normal (1)
1	c.56-151T>C	–	–	–	–	–	Normal (1)
1	c.56-62T>C	–	–	–	–	–	Normal (1)
2	c.87+1G>A	dss 79.8→0	dss 8.3→0	dss 0.9→0	dss 0.6→0	dss 84.1→0	Complete exon 2 skipping
2	c.87+26T>C	–	–	–	–	–	Normal (1)
**2**	**c.87+363A>G**	**–**	**–**	**–**	**–**	**dss 0→65.5**<br>**ass 0→83.3**	**Normal (3)**
2	c.87+705G>T	–	–	–	–	–	Normal (1)
2	c.88-559C>T	–	–	–	–	–	Normal (1)
2	c.88-352A>G	–	–	–	–	ass 72.3→0	Normal (2)
2	c.88-23A>T	–	–	–	–	–	Normal (1)
3	c.194+2T>C	dss 82.6→72.3	dss 11.1→0	dss 1.0→0	dss 4.0→0	dss 92.1→0	Partial exon 3 skipping
**3**	**c.194+13T>G**	**dss 0→82.0**	**dss 0→9.5**	**dss 0→0.9**	**–**	**dss 0→86.9**	**Normal (3)**
3	c.194+90A>T	–	–	–	–	ass 68.0→0	Normal (2)
3	c.194+184T>A	–	–	–	–	dss 73.2→0	Normal (2)
3	c.194+1159C>G	–	–	–	–	–	Normal (1)
**3**	**c.194+1504A>G**	**dss 0→77.2**	**–**	**dss 0→0.7**	**–**	**dss 0→83.2**	**Normal (3)**
3	c.195-1645G>C	–	–	–	–	–	Normal (1)
3	c.195-1570C>A	–	–	–	–	dss 0→65.3	Normal (3)
3	c.195-1538C>T	–	–	–	–	–	Normal (1)

3	c.195-1399G>A	dss 75.8→0	–	–	–	ass 0→79.2	Normal (3)
3	c.195-478T>G	–	–	–	–	–	Normal (1)
3	c.195-414C>T	–	–	–	–	–	Normal (1)
**3**	**c.195-323C>T**	**–**	**dss 0→6.3**	**dss 0→0.7**	**–**	**dss 0→75.1**	**Normal (3)**
3	c.195-73_72delCT	–	–	–	–	–	Normal (1)

The column shaded in grey was excluded from consideration. The two canonical splice site variants are highlighted in blue. The four variants that were selected for quantitative RT-PCR analysis in the present study are highlighted in bold. The obvious inappropriate predictions are highlighted in red. Abbreviations: dss, donor splice site; ass, acceptor splice site.

[a]In accordance with Zou et al. [2, 3].

[b]Refer to text for details.
