# Supplementary material for: In silico prioritization and further functional characterization of SPINK1 intronic variants
Source: Hum Genomics. 2017 May 4;11:7. doi: 10.1186/s40246-017-0103-9 (PMC5418720; doi:10.1186/s40246-017-0103-9)
Supplement: Supplementary file 3 — Alamut-predicted impact of the SPINK1 c.87 + 1G > 1, c.194 + 2T > C, and c.194 + 13T > G variants on the disruption or creation of splice sites. (PDF 418 kb) [file 40246_2017_103_MOESM3_ESM.pdf]

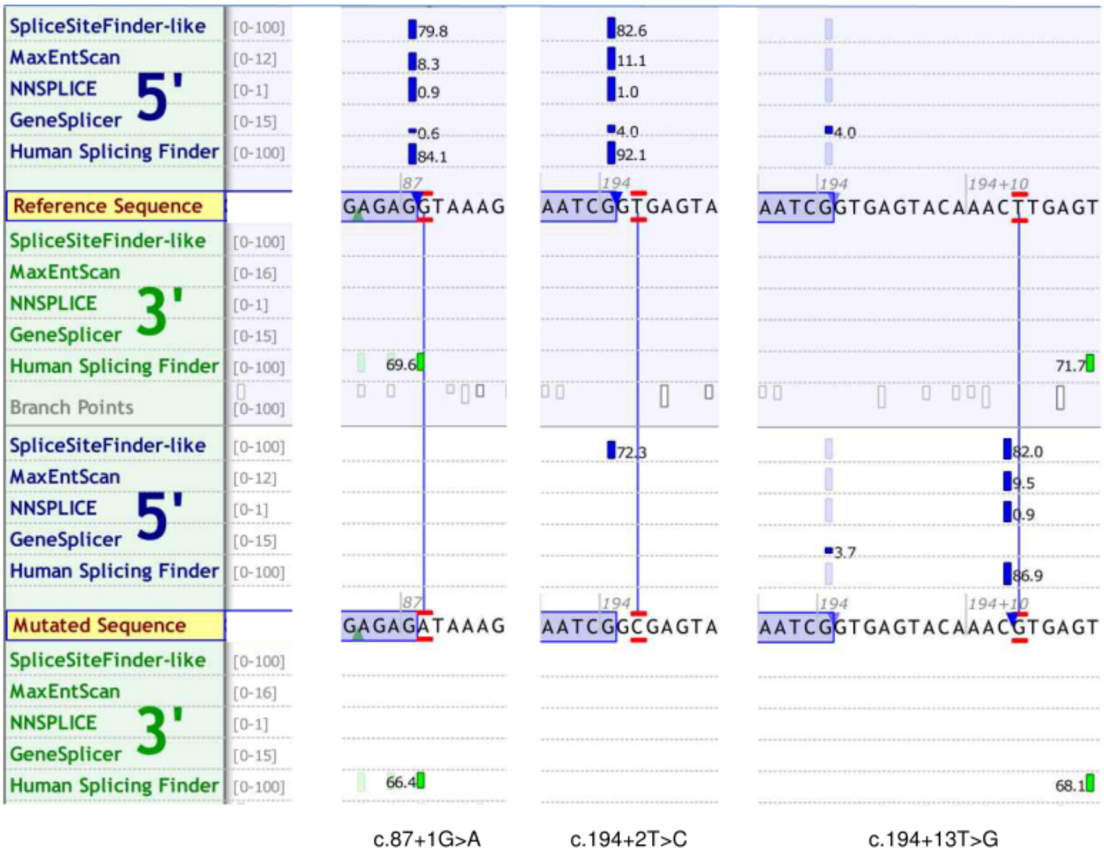

Figure S2 Alamut-predicted impact of the *SPINK1* c.87+1G>1, c.194+2T>C and c.194+13T>G variants on the disruption or creation of splice sites.
