# Supplementary material for: In silico prioritization and further functional characterization of SPINK1 intronic variants
Source: Hum Genomics. 2017 May 4;11:7. doi: 10.1186/s40246-017-0103-9 (PMC5418720; doi:10.1186/s40246-017-0103-9)
Supplement: Supplementary file 4 — Relative mRNA expression levels of the SPINK1 c.194 + 13T > G variant-containing maxigene in transfected HEK293T cells in the presence (gray) and absence (black) of cycloheximide as determined by quantitative RT-PCR analysis. (PDF 225 kb) [file 40246_2017_103_MOESM4_ESM.pdf]

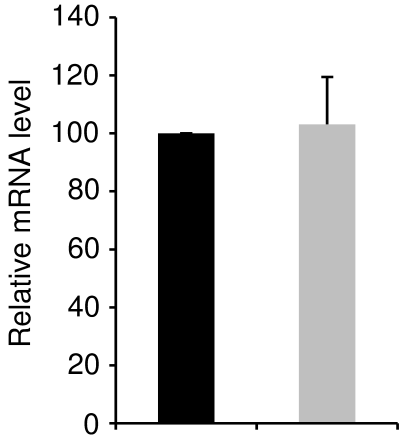

Figure S3 Relative mRNA expression levels of the *SPINK1* c.194+13T>G variant-containing maxigene in transfected HEK293T cells in the presence (gray) and absence (black) of cycloheximide as determined by quantitative RT-PCR analysis. Results are given as the mean ± SD from three independent transfection experiments.
