# Supplementary material for: In silico prioritization and further functional characterization of SPINK1 intronic variants
Source: Hum Genomics. 2017 May 4;11:7. doi: 10.1186/s40246-017-0103-9 (PMC5418720; doi:10.1186/s40246-017-0103-9)
Supplement: Supplementary file 5 — Further analyses of the SPINK1 c.194 + 13T > G variant in a minigene assay. (PDF 184 kb) [file 40246_2017_103_MOESM5_ESM.pdf]

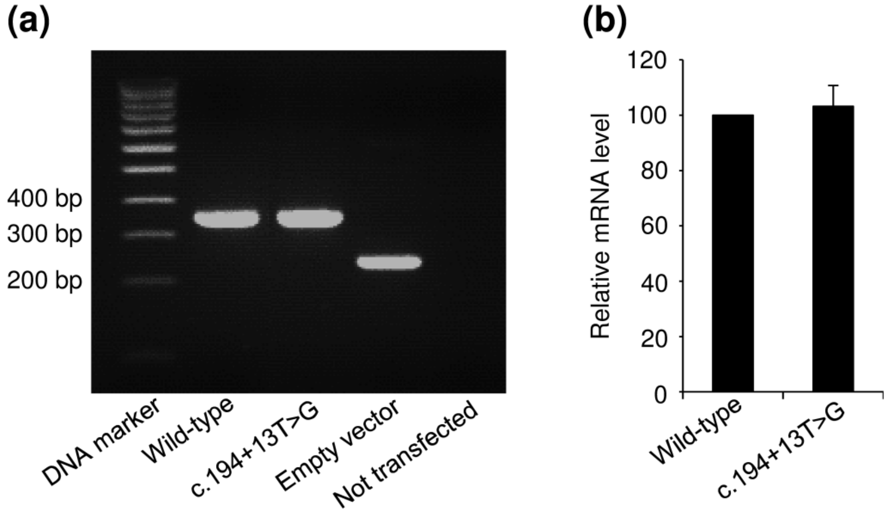

Figure S4. Further analyses of the *SPINK1* c.194+13T>G variant in a minigene assay. **a** RT-PCR analyses of HEK293T cells transfected with minigene expression vectors carrying the wild-type and c.194+13T>G variant. Empty vector, the Exontrap vector pET01. **b** Relative mRNA expression level of the c.194+13T>G variant compared to that of the wild-type as determined by quantitative RT-PCR analysis of HEK293T cells transfected with the corresponding minigene expression constructs. Results are given as the mean ± SD from three independent transfection experiments.
